# Endovascular Treatment for Aneurysms Located in the Posterior Communicating Artery (PCoA) by the Swinging-Tail Technique: A Technical Note

**DOI:** 10.3390/jcm11195955

**Published:** 2022-10-09

**Authors:** Jiejun Wang, Longhui Zhang, Linggen Dong, Shuai Zhang, Haoyu Zhu, Chuhan Jiang, Ming Lv

**Affiliations:** 1 Department of Neurosurgery, Beijing Tiantan Hospital, Capital Medical University, Beijing 100070, China; 2 Department of Interventional Neuroradiology, Beijing Neurosurgical Institute and Beijing Tiantan Hospital, Capital Medical University, Beijing 100070, China; 3 Department of Neurosurgery, Beijing Jingmei Group General Hospital, Beijing 100042, China

**Keywords:** endovascular treatment, aneurysms, posterior communicating artery (PCoA), swinging-tail technique

## Abstract

Purposes: A stent-assisted coil (SAC) is a safe and effective treatment modality for some complex intracranial aneurysms, especially for wide neck aneurysms. However, some wide neck aneurysms with a tough angle and located in the posterior communicating artery (PCoA) are challenging to treat with a SAC. This study aimed to examine and discuss the swinging-tail technique for treating wide neck aneurysms located in the PCoA using a SAC by Prof. Lv. Materials and Methods: We retrospectively reviewed our institutional clinical database and identified nine patients with neck aneurysms located in the PCoA, and these patients underwent the swinging-tail technique by Prof. Lv, which is a novel technique of releasing a stent, from June 2016 to September 2021. Results: In this study, nine patients underwent SAC treatment using the swinging-tail technique by Prof. Lv. Aneurysmal complete occlusion was observed in every patient without any complications, as shown by immediate postoperative angiography. Additionally, the modified Rankin scale was monitored for clinical outcomes in the follow-up. One patient died postoperatively due to severe SAH with an intraventricular hemorrhage. Four of nine patients underwent imaging follow-up, demonstrating the complete occlusion of aneurysms; eight patients underwent clinical follow-up and achieved a favorable clinical outcome (modified Rankin scale score: 0–2). Conclusion: The SAC treatment for wide neck aneurysms located in the PCoA can be challenging for operators because of the specific location, resulting in inadequate vessel wall apposition by antegrade stenting via the ipsilateral vessel. In this circumstance, the swinging-tail technique may be a feasible and effective choice.

## 1. Introduction

Endovascular stents are widely used during the embolization of intracranial aneurysms. Theoretically, stent deployment provides a mechanical scaffold for coils inside an aneurysm, allowing improved packing density and neck coverage [1]. When treating intracranial aneurysms, stents have the advantage of reducing the recurrence rate, improving the progressive occlusion rate, and enhancing the long-term prognosis [2]. Therefore, stent-assisted coils (SACs), a type of endovascular therapeutic modality, have been increasingly used to treat complex intracranial aneurysms, including wide-necked aneurysms, with excellent effectiveness and safety [3]. However, a type of rare but refractory lesion exists, located in the posterior communicating artery (PCoA), which can be challenging to treat when using SACs [4,5,6]. The conventional endovascular treatment modality (antegrade stenting via the ipsilateral vessel) may not achieve the satisfying outcome of allowing the stent to sufficiently cover the aneurysmal neck with a high recurrence rate [7]. Because of concerns regarding the complete coverage of the aneurysmal neck, the swinging-tail technique via the ipsilateral vessel may be a feasible and effective alternative endovascular treatment modality for aneurysms located in the PCoA. In this study, we examined and discussed our therapeutic experience in treating these refractory aneurysms located in the PCoA using the swinging-tail technique by Prof. Lv.

## 2. Materials and Methods

This retrospective study was reviewed by Beijing Tiantan hospital’s institutional review board and approved by the ethics committee of Beijing Tiantan Hospital. The patients provided their written informed consent to participate in this study.

### 2.1. Patient Population

In this study, we retrospectively reviewed the patients diagnosed of aneurysms located in the PCoA—confirmed by digital subtraction angiography (DSA) and/or computed tomography angiography (CTA)—and underwent endovascular treatment by Prof. Lv, who has sufficient clinical experience in the treatment of intracranial aneurysms using SACs, from June 2016 to September 2021. Initially, according to preoperational multidisciplinary discussions, preferences for microinvasive procedures were conveyed by the patients’ relatives; for the native characteristic of a wide neck aneurysm, endovascular treatment by SAC was regarded as the first line of treatment for the patients. Furthermore, nine patients who underwent SAC treatment using the swinging-tail technique by Prof. Lv were recruited for this study.

### 2.2. Antiplatelet and Anticoagulation Treatments

Patients without a hemorrhage were premedicated with a dual antiplatelet regimen (75 mg of clopidogrel and 100 mg of aspirin, daily) for 5 days before treatment. Patients with a hemorrhage were administered a loading dose of 300 mg of aspirin and 300 mg of clopidogrel, 2 h preoperatively. Intraoperatively, an intravenous bolus dose of heparin (100 IU/kg) was administered. Heparinization was continued to maintain an activated clotting time two- to three-fold greater than the baseline value throughout the procedure. Dual antiplatelet therapy was continued for 3 months postoperatively, and aspirin was continued for life, to prevent the formation of thrombi in the stents.

### 2.3. The Choice of Endovascular Treatment Modalities

**Figure 1:**
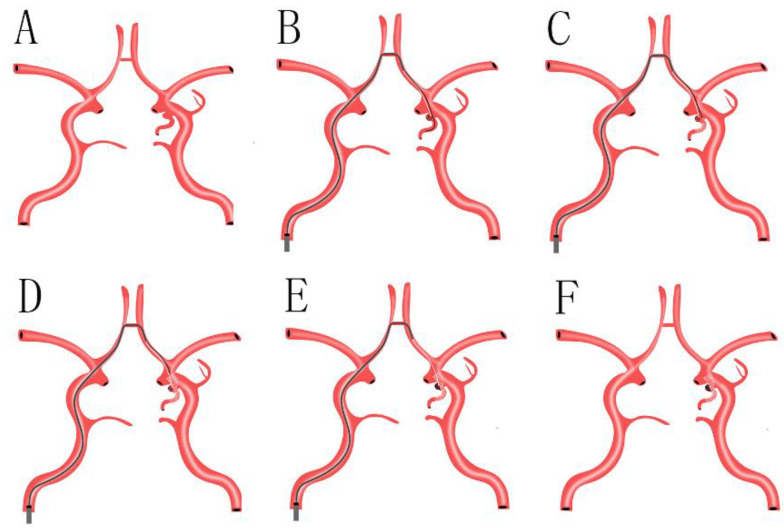
(**A**): A suitable stent was selected, according to the precise aneurysmal measurements and the diameter of parent arteries, as assessed by intraprocedural DSA; (**B**): a suitable stent microcatheter was navigated to the ipsilateral PCoA, and a suitable coil microcatheter was navigated to the aneurysmal lumen and transported several coils loosely, via the contralateral ICA and ACA and ipsilateral ICA; (**C**): the stent microcatheter was withdrawn, and the stent was gradually released in the ipsilateral PCoA; (**D**): the stent microcatheter was withdrawn continuously until the stent covered the aneurysmal neck and was released in the dismal segment of the ipsilateral ICA completely; the good wall apposition of the stent was confirmed by intraprocedural DSA, and was used to remove the stent microcatheter from the guiding catheter; (**E**): to transport several coils via a suitable coil microcatheter with the assistance of the stent, and to accomplish the complete occlusion of an aneurysm confirmed by intraoperative DSA; (**F**): to withdraw the coil microcatheter and guiding catheter from the vessel. DSA: digital subtraction angiography; ICA: internal carotid artery; ACA: anterior communicating artery; PCoA: posterior communicating artery.

**Figure 2:**
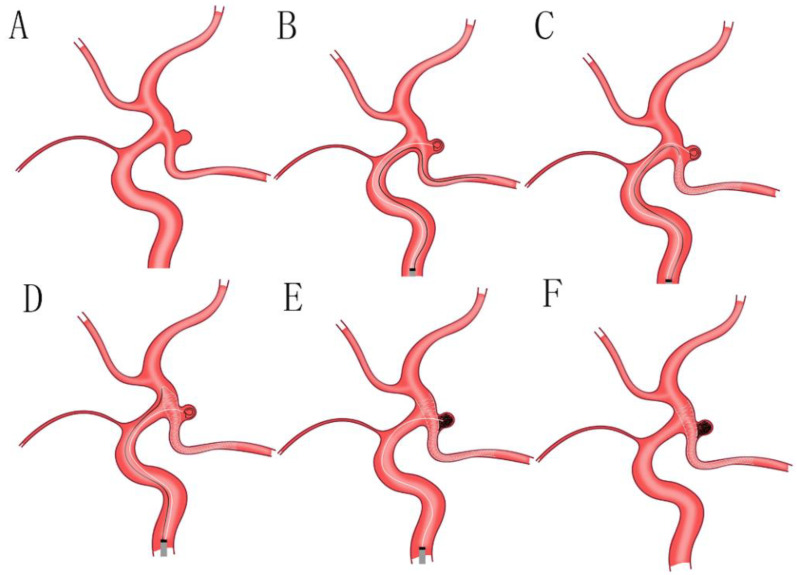
(**A**): A suitable stent was selected, according to the characteristics of aneurysms and the parameter of parent arteries, as assessed by intraprocedural DSA; (**B**): a suitable stent microcatheter was navigated to the ipsilateral PCoA, and a suitable coil microcatheter was navigated to the aneurysmal lumen and transported several coils loosely, via the ipsilateral ICA; (**C**): the stent microcatheter was withdrawn, and the stent was gradually released in the PCoA until the tip of the stent microcatheter arrived at the ostium of the PCoA in ipsilateral ICA; (**D**): the stent microcatheter was pushed, and the stent was released in the distal segment of the ipsilateral ICA completely, with complete coverage of the aneurysmal neck; the good wall apposition of the stent was confirmed by intraprocedural DSA, and was used to remove the stent microcatheter from the guiding catheter; (**E**): to transport several coils via a suitable coil microcatheter with the assistance of stent, and to accomplish the complete occlusion of an aneurysm confirmed by intraoperative DSA; (**F**): to withdraw the coil microcatheter and guiding catheter from the vessel. DSA: digital subtraction angiography; PCoA: posterior communicating artery; ICA: internal carotid artery.

By performing the precise preoperative evaluation of aneurysmal characteristics according to DSA and/or CTA in detail, we found, differentiated from the aneurysms located in internal carotid artery (ICA) C7 segment, that the aneurysms admitted to our study primarily affect the PCoA rather than the ICA C7 segment. Furthermore, there is an acute angle between the PCoA affected by an aneurysm and the proximal segment of the ICA, which limits the choice of conventional endovascular treatment by a SAC (releasing the stent via the ipsilateral vessel from the PCoA to the proximal segment of the ICA), because of inadequate wall apposition. In this circumstance, SAC via the contralateral ICA and anterior communicating artery (ACA) may be an available choice (Figure 1). However, this method has several limitations: firstly, the Willis circle of patients may have hypoplasia or are deficient, especially for the ACA, and it can result in the failure of treatment directly; secondly, this method increases the difficulty of choosing a target vessel and the incidence of vascular dissection; thirdly, this method makes the length of an operative path longer than a SAC via the ipsilateral vessel and increases treatment-associated complications. Finally, we decided to release the stent using the swinging-tail technique via the ipsilateral vessel (Figure 2).

### 2.4. Swinging-Tail Technique

All procedures were performed with the patient under general anesthesia via the transfemoral approach, and systemic heparinization was administered after the placement of a sheath. A suitable guiding catheter was navigated to the target location via a feasible procedural course. Three-dimensional rotational DSA was performed to determine the best working projection and to measure the size of the aneurysms, the diameter of the ICA and PCoA, the angle between the PCoA affected by aneurysms, and the proximal segment of the ICA (below the ostium of the PCoA). Suitable stents, with a high metal coverage rate in diseased vessels, were selected according to the aneurysmal measurements, the diameter of the PCoA, and the proximal segment of the ICA, with the aim of achieving complete coverage of the aneurysmal neck and a good wall apposition between the stent, PCoA, and the segment of the ICA. We used suitable coils to completely occlude the aneurysmal sac with the assistance of stents.

**Figure 3:**
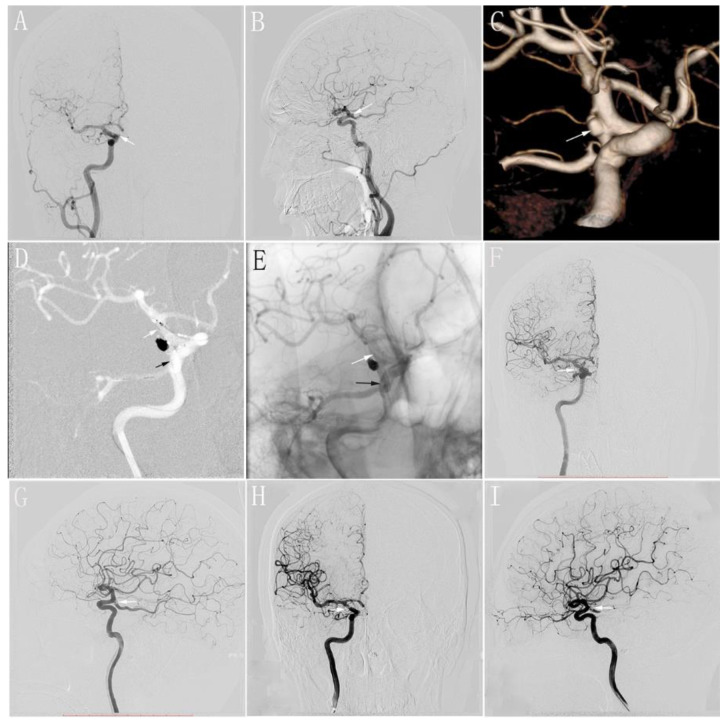
(**A**,**B**): Preoperative anteroposterior (**A**) and lateral (**B**) DSA of the R-ICA showing a wide-necked aneurysm that is located in the origin of the PCoA (white arrow); (**C**): Preoperative three-dimensional reconstruction of the R-ICA showing a wide-necked aneurysm (white arrow); (**D**): The stent is completely released by the swinging-tail technique. The distal segment of the stent is located in the PCoA (black arrow) and the proximal segment of the stent is located in the distal segment of the R-ICA at the ostium of the PCoA (white arrow); (**E**): Postoperative immediate unsubtracted DSA showing the location of stent (black arrow: the distal segment of the stent; white arrow: the proximal segment of the stent); (**F**,**G**): Postoperative immediate anteroposterior (**F**) and lateral (**G**) DSA of the R-ICA showing complete occlusion of the aneurysm (white arrow); (**H**,**I**): Anteroposterior (**F**) and lateral (**G**) DSA at 6 months of follow-up postoperatively of the R-ICA showing complete occlusion of the aneurysm (white arrow). DSA: digital subtraction angiography; PCoA: posterior communicating artery; ICA: internal carotid artery.

The points of the swinging-tail technique are as follows: (1) A suitable stent was selected according to the precise aneurysmal measurements, the diameter of the PCoA, and the proximal segment of the ICA, as assessed by intraprocedural DSA. A suitable stent is of importance for reducing the possibility of aneurysmal recurrence (Figure 2A); (2) A suitable stent microcatheter was navigated to the ipsilateral PCoA, and a suitable coil microcatheter was navigated to the aneurysmal lumen and transported several coils loosely via the ipsilateral ICA. This step requires operators to hold enough clinical experience (Figure 2B); (3) The stent microcatheter was withdrawn, and the stent was gradually released in the PCoA until the tip of the stent microcatheter arrived at the ostium of the PCoA in the ipsilateral ICA. In this circumstance, the suitable length of the stent in the microcatheter is necessary so that it can accomplish the swinging-tail technique successfully (Figure 2C); (4) The stent microcatheter was pushed, and the stent was released in the distal segment of the ipsilateral ICA completely, with complete coverage of the aneurysmal neck. This operation is also a key step, and a suitable length of the stent in the distal segment of the ipsilateral ICA is vital because a short length of stent may induce the failure of the swinging-tail technique and a long length of stent may increase the operative difficulty of this technique. The good wall apposition of the stent was confirmed by an intraprocedural DSA and was used to withdraw the stent microcatheter from the guiding catheter (Figure 2D); (5) Several coils were transported via a suitable coil microcatheter with the assistance of the stent, and the accomplished complete occlusion of an aneurysm was confirmed by an intraoperative DSA (Figure 2E); (6) The coil microcatheter was withdrawn from the guiding catheter and the guiding catheter was finally removed from vessel (Figure 2F).

The key point is that a suitable length of the stent segment released in the ipsilateral ICA was estimated; this was important because an unstable stent situation in the blood flow was ascribed to a short length of stent segment, and the failure of the swinging-tail technique was ascribed to a long length of stent segment.

## 3. Results

### 3.1. Patient and Aneurysmal Characteristics

This case series included nine patients with nine aneurysms who underwent the swinging-tail technique (female: male, 1:2), with a mean (±standard deviation) age of 62.8 ± 9.7 years (age range, 48–79 years). Two patients were diagnosed with a subarachnoid hemorrhage (SAH) by preoperative computed tomography (CT). Nine aneurysms were located in the PCoA, as confirmed by preoperative DSA. The patients’ demographics and aneurysmal characteristics are shown in Table 1 and Table 2.

### 3.2. Technical Success and Immediate Angiographic Results

In this study, all patients tolerated the procedure well, without any intraprocedural complications. Nine patients with nine aneurysms underwent endovascular treatment (SACs) using the swinging-tail technique by Prof. Lv. Additionally, all patients successfully underwent SACs using the swinging-tail technique (Figure 3). To reduce the recurrence rate of aneurysms, we aimed to achieve complete occlusion of the aneurysmal sac, with the assistance of the stent. All patients achieved complete occlusion of the aneurysm, as shown by an immediate postoperative angiography.

## 4. Clinical and Imaging Follow-Up

### 4.1. Clinical Follow-Up

Clinical follow-up data were available for all of the patients (mean follow-up, 20.1 ± 22.3 months; range, 3–66 months) by enrolling the patients or communicating via telephone. In our study, we selected the modified Rankin scale (mRS) score as our standard criteria for evaluating the clinical conditions of every patient. Preoperatively, two (2/9, 22.2%) patients were diagnosed with SAH by preoperative CT; they had relatively poor clinical conditions, with mRS scores of two and five, respectively. After undergoing treatment, three (3/9, 33.3%) patients showed improvements in their clinical conditions, three patients (3/9, 33.3%) were diagnosed with an intracranial aneurysm by physical examination coincidently without any associated symptoms. Two (2/9, 22.2%) patients experienced no clinical improvement and were presented with a chronic headache associated with the severe stenosis of dual vertebral arteries and cervical spondylosis, with an mRS score of one during follow-up. One patient (1/9, 11.1%) died of severe SAH with a mass intraventricular hemorrhage three days after the operation. The detailed clinical follow-up outcomes are shown in Table 1.

### 4.2. Angiographic Follow-Up

Four patients underwent a follow-up with DSA (mean follow-up, 9 ± 6 months; range, 6–18 months). Generally, we selected the final DSA follow-up of every patient as the time point used to evaluate the efficacy of the endovascular treatment. Four patients achieved the complete occlusion of aneurysms, as confirmed by the results of the DSA follow-up. DSA follow-up data are shown in Table 2.

## 5. Discussion

Currently, endovascular embolization is recommended as the first choice for the treatment of most intracranial aneurysms due to the advanced technique associated with endovascular embolization; this is especially evident for the application of the stent-assisted coiling technique, combined with the advantages of minimal invasiveness and a quick recovery [8]. Stents may alter hemodynamics and promote endothelial proliferation and vascular reconstruction [9]. Stents also play major roles in increasing the progressive occlusion rate and improving long-term prognoses. Aneurysmal recurrence following endovascular coil embolization can be affected by case selection and the locations of aneurysms [2]. A higher recurrence rate may be associated with poorer immediate embolization results, increased aneurysmal neck exposure to blood flow, and sustained impact [10]. Therefore, the selection of the appropriate stent deployment strategy is important for reducing the exposure area of the aneurysmal neck to blood flow. Furthermore, an appropriate stent deployment strategy is important to ensure the complete coverage of the aneurysmal neck by stents and to further reduce the recurrence rate of aneurysms. Different stenting techniques, such as Y-stenting, the waffle cone technique, and horizontal stenting, have been developed for complex aneurysms [11]. An aneurysm located in the PCoA is a type of intracranial, complex aneurysm, with a lower incidence than other intracranial aneurysms, and without an effective and feasible endovascular therapeutic strategy [8]. Therefore, examining and discussing the potential endovascular treatments for aneurysms located in PCoA would be useful.

Because these lesions are rare and differentiated from the aneurysms of the posterior communicating segment of the distal ICA, their anatomical relationships have been poorly described. Similarly, reports concerning the endovascular treatment of these lesions are rare, and the technical aspects are poorly detailed [12]. Aneurysms located in the PCoA primarily affect the PCoA instead of the ICA, which makes therapeutic decisions complicated. Takashi et al. reported that patients with an aneurysm of the PCoA underwent successful endovascular treatment by the complete occlusion of the PCoA [13]. These patients obtained excellent clinical improvements without conspicuous neurological deficits. Although some patients benefit from sufficient collateral circulation which is necessary to avoid ischemic events following the sacrifice of arterial branches, predicting which patients are at risk of procedural-related ischemic events can be difficult [5]. Munarriz [14] suggested that hemodynamic stress might be important for aneurysms located in the PCoA because these aneurysms are often associated with a dominant PCoA (fetal type), in which the PCoA is the major blood supply to the posterior cerebral artery, consistent with a higher flow through this artery. The preservation of the PCoA is important, even if it is hypoplastic. Therefore, the primary target during the endovascular treatment of aneurysms located in the PCoA is the complete occlusion of the aneurysm, while preserving the parent vessel and perforators. However, the most feasible and effective endovascular treatment modality for achieving the primary target during the treatment for these lesions remains unknown.

In our study, we found that all aneurysms were wide-necked aneurysms (neck diameters >4 mm or a dome-to-neck ratio <2), and the angle between the PCoA affected by aneurysms and the proximal segment of the ICA was acute. When evaluating the technical feasibility, Prof. Lv, an experienced neuro-interventional expert, believed that conventional endovascular treatment (antegrade stenting through an ipsilateral vessel) would not be able to achieve excellent therapeutic outcomes, with a large possibility of recurrence. Recently, for this complex lesion, Yang et al. reported the novel proximal-end stenting technique, which uses the proximal end of the stent to assist with the embolization of a wide-necked aneurysm located in the PCoA [11]. However, one of the limitations of this method is that a stent in the dismal segment of the ICA has no function, neither assisting the coiling nor protecting the patency of the arterial branch. Additionally, the malapposition of stents may increase the risks of serious adverse events, such as thromboembolic complications [15]. Although the aneurysmal neck is not located in the ICA, we aimed to develop an endovascular therapeutic strategy in which the implant covers the aneurysmal neck completely, leading to intra-aneurysmal thrombosis due to a reduced blood flow in the aneurysm sac, thus, preserving the patency of the branch.

According to the preoperative DSA performed in every patient, we initially rejected the conventional endovascular treatment modality (antegrade stenting through the ipsilateral vessel) because of the particular location of the aneurysm and the acute angle between the PCoA and the proximal segment of the ICA. Yang et al. [8] reported that, for the SAC treatment of an aneurysm located in the PCoA, either the ipsilateral or contralateral vessel approach may be used, depending on the condition of the vascular structure. In our study, we thought that antegrade stenting via the ipsilateral vessel was preferred when the angle between the PCoA affected by aneurysms and the proximal segment of the ICA was obtuse. However, retrograde stenting via the contralateral vessel is used in cases with an acute angle between the PCoA and the proximal segment of the ICA. However, retrograde stenting via the contralateral vessel involves more operative risks in the cases of increasing the length of the operative pathway and the difficulty of choosing a target vessel. Meanwhile, retrograde stenting via a contralateral vessel is not suitable for some patients with hypoplasia or a deficient anatomical structure of the circle of Willis.

Additionally, although these lesions could not be effectively managed by conventional antegrade stenting via the ipsilateral vessel, we developed a novel technique via the ipsilateral vessel (the swinging-tail technique) to treat these complex lesions. The swinging-tail technique is a procedure where the stent is released from the PCoA affected by an aneurysm to the distal segment of the ICA via the ipsilateral ICA. In this situation, the distal end of the stent is located in the PCoA affected by an aneurysm, and the proximal end of the stent is located in the distal segment of the ipsilateral ICA, at the ostium of the PCoA. The primary advantage of this technique is that the aneurysmal neck is completely covered by the stent, and the ipsilateral PCoA is simultaneously preserved to the utmost extent, with a low possibility of recurrence and ischemic events. Fortunately, in this study, all patients obtained excellent clinical outcomes according to the results of the clinical follow-up, except the patient with a poor preoperative condition who died three days after treatment due to a severe SAH with a mass intraventricular hemorrhage; all aneurysms that underwent the treatment of the swinging-tail technique achieved complete occlusion intraoperatively and four aneurysms had no signs of recurrence according to intraoperative DSA and DSA follow-up, respectively. Similarly, retrograde stenting via the contralateral vessel can accomplish the same therapeutic outcome as the swinging-tail technique in the condition of satisfying the anatomic vascular structure. The swinging-tail technique requires a greater technical ability for operators so that it is not regarded as a normal treatment modality for this kind of refractory lesion. However, according to our treatment strategies for the aneurysms located in the PCoA, the swinging-tail technique can be an alternative treatment modality.

## 6. Limitations

This study has several limitations. First, this was a retrospective study, with a relatively small number of patients. Second, the swinging-tail technique, as a novel technique for treating aneurysms located in the PCoA, requires the increased technical ability of operators. Therefore, popularizing this technique for the treatment of the aneurysms located in the PCoA will be difficult and time-consuming, which precludes the precise therapeutic evaluation of this technique for these complex lesions. Third, although our patients underwent endovascular treatments—with complete occlusion of the aneurysms, as confirmed by immediate postoperative angiographies—long-term follow-ups remain necessary, and we only received the DSA follow-up of four patients. Therefore, a long-term study involving a large cohort is required to obtain sufficient evidence to evaluate the effectiveness and safety of the swinging-tail technique for the treatment of aneurysms located in the PCoA.

## 7. Conclusions

An aneurysm located in the PCoA is challenging for SAC treatments because of the specific location between the PCoA and ICA. The PCoA affected by aneurysms and the proximal segment of the ICA form an acute angle, which makes the apposition of stents for an aneurysmal neck insufficient, resulting in increasing the possibility of the recurrence of aneurysms. In this circumstance, the swinging-tail technique, via the ipsilateral vessel, is a feasible and effective choice, though requiring the increased technical ability of operators. To evaluate the effectiveness and safety of the swinging-tail technique, a long-term clinical and/or DSA follow-up is required for these patients.

## Figures and Tables

**Table 1:** Patients’ Demographics.

Case	Age (Y) /Sex	Hypertension	Diabetes	Symptoms	Pre-mRS	Pre-CT	H-H	Modified Fisher Scale	Intraprocedural Complication	Postoperative Complication	Clinical-FU Period/M	mRS-FU
1	65/M	Y	Y	No symptoms	1	NA	-	-	N	N	66	0
2	49/M	Y	N	Chronic headache	1	NA	-	-	N	N	42	1
3	59/F	Y	Y	Acute headache	1	No signs	-	-	N	N	16	0
4	68/F	N	N	Sudden severe headache	2	SAH	2	2	N	N	14	0
5	79/F	N	Y	Sudden severe headache	5	SAH	4	4	N	N	-	6
6	66/F	Y	N	Sudden severe headache	1	No signs	-	-	N	N	8	1
7	68/F	Y	N	No symptoms	0	No signs	-	-	N	N	8	0
8	63/F	Y	N	No symptoms	0	NA	-	-	N	N	4	0
9	48/M	N	N	No symptoms	0	No signs	-	-	N	N	3	0

NA: not available; pre-mRS: preoperative modified Rankin scale; pre-CT: preoperative computed tomography; H-H: Hunt–Hess scale; FU: follow-up; SAH: subarachnoid hemorrhage; Y: Yes; N: No; M: Male; F: Female.

**Table 2:** Aneurysmal Characteristics.

Case	Side	AN/mm	DNR	# The Angle/Degree	TM	Stent	Size of Stent (mm)	S.N	Immediate DSA Postoperatively	DSA FU Period/M	DSA FU
1	R	4.06	1.23	75.6	SAC	LVIS	3.5 × 15	1	Complete occlusion	18	Complete occlusion
2	R	3.60	1.11	62.2	SAC	LVIS	3.5 × 15	1	Complete occlusion	6	Complete occlusion
3	R	3.50	1.00	24.3	SAC	LEO + Baby	3.2 × 16	1	Complete occlusion	6	Complete occlusion
4	L	6.66	1.13	75.1	SAC	Neuroform Atlas	3.0 × 21	1	Complete occlusion	6	Complete occlusion
5	L	4.10	0.98	35.5	SAC	LEO + Baby	2.5 × 18	1	Complete occlusion	-	-
6	L	4.23	0.82	32.1	SAC	Neuroform Atlas	3.0 × 15	1	Complete occlusion	-	-
7	L	5.37	0.73	35.0	SAC	Neuroform Atlas	3.0 × 21	1	Complete occlusion	-	-
8	L	5.00	0.20	37.6	SAC	Neuroform Atlas	3.0 × 15	1	Complete occlusion	-	-
9	L	2.44	0.66	58.9	SAC	Neuroform Atlas	4.0 × 15	1	Complete occlusion	-	-

# The angle between the posterior communicating artery affected by aneurysm and the proximal segment of ipsilateral ICA. AN: diameter of the aneurysmal neck; DNR: dome-to-neck ratio; TM: treatment modality; S.N: number of stents; FU: follow-up; R: Right; L: Left; SAC: stent-assisted coiling technique; DSA: digital subtraction angiography.

## Data Availability

The data presented in this study are available on request from the corresponding author. The data are not publicly available due to patients’ privacy.
